# E-Cigarettes: Are They as Safe as the Public Thinks?

**DOI:** 10.6004/jadpro.2016.7.2.9

**Published:** 2016-03-01

**Authors:** Wendy H. Vogel

**Affiliations:** Wellmont Cancer Institute, Kingsport, Tennessee

An important part of our practice in oncology is assisting patients in smoking cessation and providing them with information about factors that increase their cancer risk. Smoking accounts for almost 90% of all lung cancers and as much as 30% of all cancer deaths (Centers for Disease Control and Prevention [CDC], 2014). As advanced practitioners, we can influence the incidence, disease outcomes, and mortality rates of cancer by promoting smoking cessation. Over the past 20 years, smoking cessation programs and pharmacologic agents have been utilized to aid in tobacco abstinence. It is well understood that a combination of cognitive behavioral and psychological counseling plus pharmacologic agents provides the highest abstinence rates (Karam-Habe, Cinciripini, & Gritz, 2014).

Electronic cigarettes (e-cigarettes)—often referred to as vapes, vape pens, or hookah pens—have been touted as a method of smoking cessation. These small, battery-operated devices use an electric heater that aerosolizes liquid nicotine (Cobb, Hendricks, & Eissenberg, 2015). This liquid usually contains other compounds such as propylene glycol, vegetable glycerin, and flavorants. The amount of nicotine varies from much less to more nicotine when compared to regular cigarettes. These devices may or may not look like cigarettes; some may even look like a lighter or a pen, potentially enabling users to hide their nicotine use.

## HISTORY

In the United States, the use of e-cigarettes has been growing since they were first introduced into the market in 2007. A study among college students in Texas given online surveys 14 months apart noted a doubling in e-cigarette use in current cigarette smokers and a tripling of use in those who did not smoke cigarettes (Loukas, Batanova, Fernandez, & Agarwal, 2015). The CDC reported that e-cigarette use tripled in middle and high schoolers from 2011 to 2013 (US Department of Health and Human Services, 2013). Data from the annual Florida Youth Tobacco Survey noted that the use of e-cigarettes tripled from 2011 to 2014 (Porter et al., 2015) in middle and high school students. These devices are widely marketed through television, the Internet (sites such as YouTube), and social media. Flavorings and attractive packaging add to their appeal.

Caucasians, males, younger people, and those with higher incomes are more likely to be users of e-cigarettes (Schraufnagel, 2015). Older people are more likely to use e-cigarettes as a smoking cessation aid than are younger people (Born et al., 2015). However, statistics on the use of these devices may be underestimated, as users may deny using tobacco/nicotine or may not realize the chemical contents of their "vaping."

## STUDIES REGARDING CESSATION

Despite being touted as a method for smoking cessation, there are few studies that show that e-cigarettes actually do assist in decreasing smoking (Sutfin, Reboussin, Debinski, Wagoner, Spangler, & Wolfson, 2015). In fact, some studies indicate that they may contribute to nicotine addiction (Fillon, 2015). A study performed with college students in North Carolina and Virginia found that e-cigarettes did not deter cigarette smoking and may have actually contributed to continued smoking. The California Longitudinal Smokers Study noted that a survey of female, obese, current cigarette smokers who used e-cigarettes did not see a reduction in their use of cigarettes or a decreased dependence on cigarettes (Strong et al., 2015). Other studies have also not found e-cigarettes to be useful in smoking cessation (Brose, Hitchman, Brown, West, & McNeill, 2015). In the cancer population, the use of e-cigarettes does not appear to decrease rates of smoking (Borderud, Li, Burkhalter, Sheffer, & Ostroff, 2014).

A recent Cochrane review concluded that there is evidence for the use of e-cigarettes in smoking cessation and abstinence for at least 6 months, but the quality of the studies in question was rated as "low" by GRADE standards (McRobbie, Bullen, Hartmann-Boyce, & Hajek, 2014). For reference, the "low" grade was given due to the small number of trials, low event rates, and wide confidence intervals. When evaluating existing literature on the efficacy and safety of e-cigarettes, health-care professionals note many methodologic issues, severe conflicts of interests, paucity of studies, small studies, inconsistencies and contradictory results and lack of long-term follow-up (Pisinger & Dossing, 2014).

## RISKS AND SAFETY

The safety of e-cigarettes has yet to be established. Many health-care providers are unaware of their potential risks (Crowley, 2015). Despite widespread promotion that e-cigarettes are safer than regular cigarettes due to less tar production, there are multiple chemicals found in these products. These include nicotine, formaldehyde, acetaldehyde, lead, acetone, copper, and cadmium (Cheng, 2014; Jensen, Luo, Pankow, Strongin, & Peyton, 2015; Lerner et al., 2015; Varlet et al., 2015). These compounds can be addictive and/or carcinogenic. For example, formaldehyde is an International Agency for Research on Cancer group 1 carcinogen. The amount of toxins to which a user is exposed varies between brands and even within the same brand.

Studies on the acute effects of e-cigarette use are available (Table 1), but data on long-term effects are lacking (Battista et al., 2013; Farsalinos et al., 2015). Table 2 lists several potential harmful effects of e-cigarettes. Nicotine is known to be a highly addictive carcinogen. It affects multiple body systems and is especially harmful to the developing brain and other organs. Nicotine has both stimulating and desensitizing receptors that may have erratic effects (Schraufnagel, 2015). Nicotine exposure increases the risk of cardiovascular, respiratory, and gastrointestinal disorders, including cancers (Mishra, Chaturvedi, Datta, Sinukumar, Joshi, & Garg, 2015).

**Table 1:**
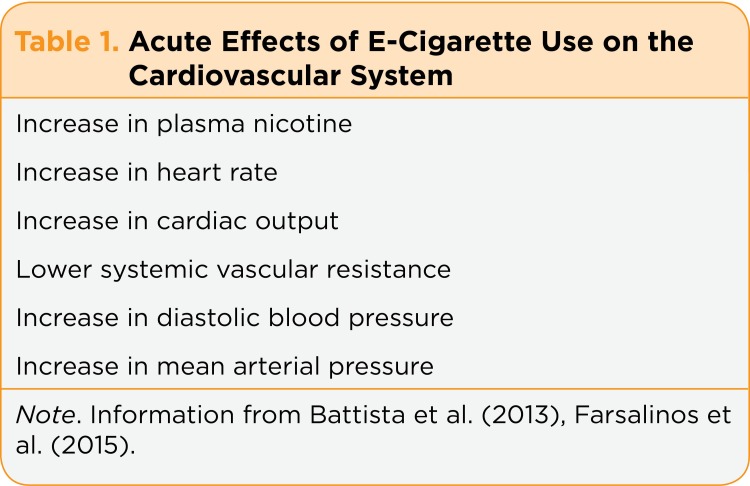
Acute Effects of E-Cigarette Use on the Cardiovascular System

**Table 2:**
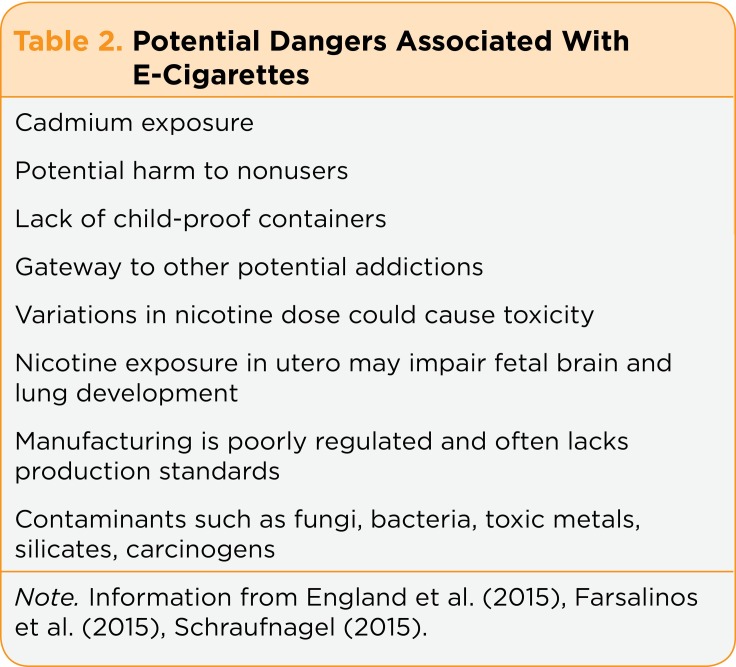
Potential Dangers Associated With E-Cigarettes

Preclinical data suggest that e-cigarettes increase the virulence of drug-resistant bacteria and decrease the ability of lung cells to destroy bacteria (Crotty Alexander et al., 2014).

There are no current studies that prove that e-cigarettes are safe. In fact, quality studies would be extremely difficult at this time due to lack of conformity between brands and even within the same brand. There is no current US regulation, production standard or quality control on these products (Nelluri, Murphy, & Mookadam, 2015; Tremblay et al., 2015). In fact, e-cigarettes are not licensed as drug or tobacco products (Schraufnagel, 2015). A recent study of nicotine concentrations in 91 e-cigarette liquids in the United States, Poland, and South Korea found that 19% of these products had significant discrepancies between the labeled content amount and the actual amount of nicotine. In three of the US products, nicotine was found in products that were labeled "nicotine-free" (Goniewicz et al., 2015).

Another safety concern associated with e-cigarettes is the growing numbers of accidental poisonings in children from e-cigarette liquid (Chatham-Stephens et al., 2014). Symptoms of e-cigarette poisoning are tachycardia, tremor, chest pain, and hypertension. This can progress to altered mental status, bradycardia, hypotension, nausea, respiratory paralysis, atrial fibrillation, and dyspnea (Nelluri, Murphy, & Mookadam, 2015; Normandin & Benotti, 2015). Replacement containers of liquid nicotine are often not child resistant. It may also have bright packaging and inviting flavors such as gummy bear, cotton candy, sweet tarts, chocolate, and donut. As little as 1 teaspoon of liquid nicotine could be fatal in children (Normandin & Benotti, 2015).

## REGULATION

Debate continues regarding the regulation of e-cigarettes. In the United States, there are various regulations about access by minors, use in certain venues, licensure, marketing, and taxation (Marynak et al., 2014; Tremblay et al., 2015). The FDA is in the process of finalizing a rule that would extend its authority to products such as e-cigarettes, requiring that such devices/products be registered with the FDA and only marketed after FDA review (FDA, 2014). Under this rule, manufacturers could not make any direct or indirect claims of reduced risk of cancer unless there is scientific evidence that supports the claim. Free samples would be prohibited, and there would be minimum age and identification restrictions to prevent sale to minors. This proposed rule is being vigorously fought in Congress at this time.

Complicating the regulation of e-cigarettes is the fact that the sale of these devices is quite profitable. In the United States, in 2012 the market was estimated by Wells Fargo as being a "niche" worth $300 million (Herzog, 2014). By 2013, the market was thought to be worth $2.5 billion, and will likely overtake cigarette sales within 10 years. A recent Research and Markets report noted that e-cigarette sales are expected to increase about 24.2% annually through 2018 (Research and Markets, 2014). E-cigarette brand owners are primarily large tobacco companies (such as Altria, R.J. Reynolds, and Philip Morris International). Table 3 gives a nonexhaustive list of policy statements and position papers by various professional organizations regarding FDA control over these products.

**Table 3:**
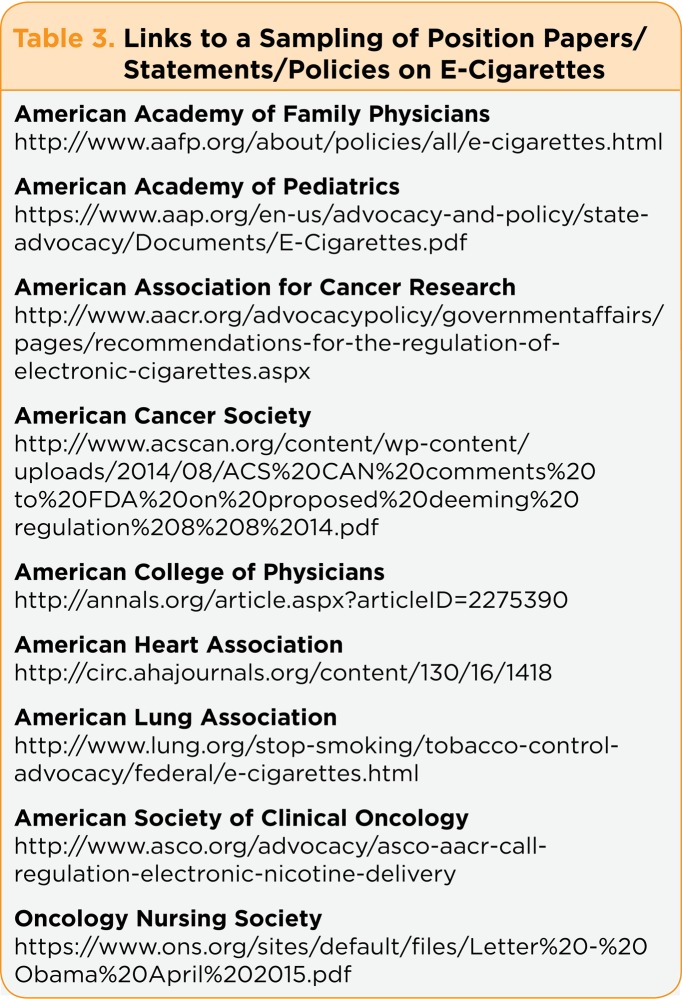
Links to a Sampling of Position Papers/ Statements/Policies on E-Cigarettes

## CONCLUSION

The oncology advanced practitioner must be able to answer questions regarding the safety and efficacy of e-cigarettes. Assisting the patient in smoking cessation will improve quality of life and decrease mortality. A patient information sheet about e-cigarettes is provided below for use in your practice.

**Figure 1:**
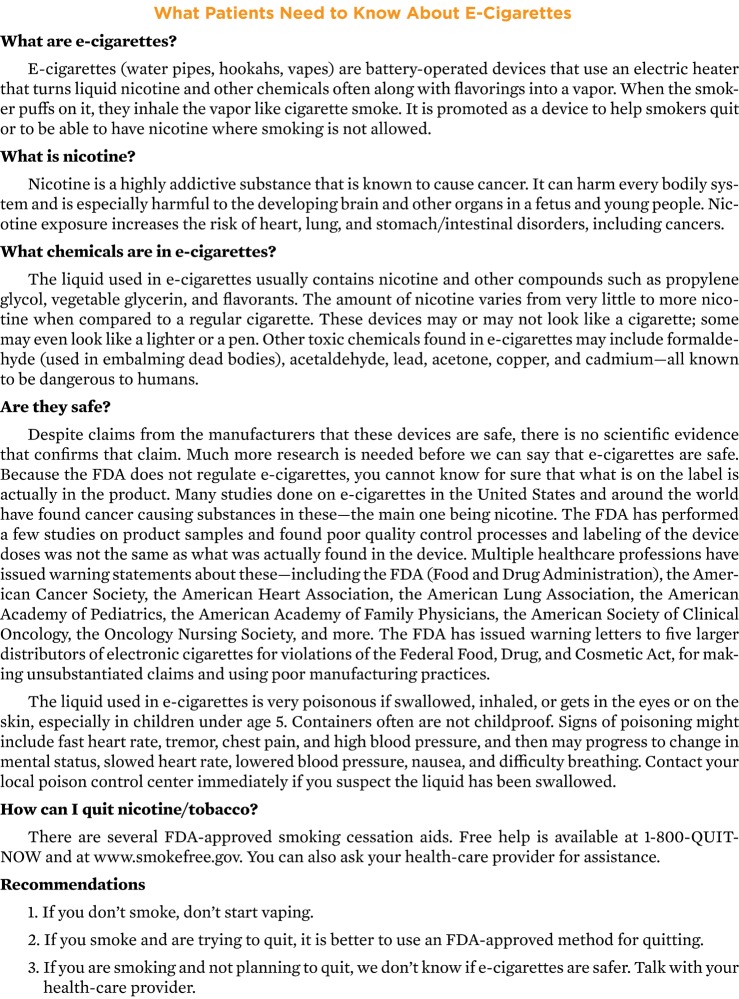
What Patients Need to Know About E-Cigarettes
